# BABY FRIENDLY HOSPITAL INITIATIVE: 25 YEARS OF EXPERIENCE IN BRAZIL

**DOI:** 10.1590/1984-0462/;2019;37;4;00004

**Published:** 2019-06-19

**Authors:** Joel Alves Lamounier, Roberto Gomes Chaves, Maria Albertina Santiago Rego, Maria Cândida Ferrarez Bouzada

**Affiliations:** a Universidade Federal de São João del-Rei, Campus Dom Bosco, São João del-Rei, MG, Brazil.; b Universidade de Itaúna, Itaúna, MG, Brazil.; c Universidade Federal de Minas Gerais, Belo Horizonte, MG, Brazil.

**Keywords:** Milk, human, Breast feeding, Brazil, Leite materno, Aleitamento materno, Brasil

## Abstract

**Objective:**

To describe the experience of the 25-year-old trajectory of the Baby Friendly Hospital Initiative (BFHI) in Brazil. The first unit was implemented in 1992.

**Methods:**

Information and data were collected from publications on the World Health Organization (WHO), the United Nations International Children’s Emergency Fund (UNICEF) and the Ministry of Health websites and in national and international journals, about the period 1990-2017. The descriptors used were: “iniciativa hospital amigo da criança”, “hospital amigo da criança”, “*baby friendly initiative hospital*”, “aleitamento materno” and “*breastfeeding*”. The number of hospitals in the 25 years, the course of the BFHI and its repercussions on breastfeeding in Brazil were evaluated.

**Results:**

The BFHI is an intervention strategy in hospital care at birth focused on the implementation of practices that promote exclusive breastfeeding from the first hours of life and with the support, among other measures of positive impact on breastfeeding, of the International Code of Marketing of Breastmilk Substitutes. Currently, the initiative has been revised, updated and expanded to integrate care for newborns in neonatal units and care for women since prenatal care. It can be concluded that, during these 25 years, the quantity of hospitals varied greatly, with numbers still below the capacity of hospital beds. BFHI shows higher rates of breastfeeding than non-accredited hospitals. However, the number of hospitals are still few when compared to other countries.

**Conclusions:**

The BFHI has contributed to breastfeeding in Brazil in recent decades. Greater support for public policies is needed to expand the number of accredited institutions in the country.

## RESUMO

**Objetivo:**

Descrever a experiência de 25 anos da Iniciativa Hospital Amigo da Criança (IHAC) no Brasil, cuja primeira unidade foi implementada em 1992.

**Métodos:**

Informações e dados foram obtidos em publicações nos *sites* da Organização Mundial da Saúde (OMS), do Fundo Internacional de Emergência para a Infância das Nações Unidas (UNICEF) e do Ministério da Saúde e em periódicos nacionais e internacionais, abrangendo o período de 1990 a 2017. Utilizaram-se os descritores: “iniciativa hospital amigo da criança”, “hospital amigo da criança”, “*baby friendly initiative hospital*”, “aleitamento materno” e “*breastfeeding*”. Foram avaliados o número de hospitais nos 25 anos, a trajetória da IHAC e suas repercussões sobre o aleitamento materno no Brasil.

**Resultados:**

A IHAC é uma estratégia de intervenção na assistência hospitalar ao nascimento com foco na implementação de práticas que promovem o aleitamento materno exclusivo desde as primeiras horas de vida e com o apoio, entre outras medidas de impacto positivo na amamentação, do Código Internacional de Comercialização de Substitutos do Leite Materno. Atualmente, a iniciativa foi revisada, atualizada e expandida para integrar o cuidado aos recém-nascidos nas unidades neonatais e na atenção à mulher desde o pré-natal. Pôde-se concluir que, ao longo desses 25 anos, a quantidade de hospitais variou muito, com números ainda aquém da capacidade de leitos hospitalares. Hospitais credenciados como o Hospital Amigo da Criança mostram índices de amamentação superiores ao de hospitais não credenciados, entretanto o número de hospitais credenciados no Brasil ainda é pouco se comparado com outros países.

**Conclusões:**

A IHAC contribuiu para o aleitamento materno no Brasil nessas últimas décadas. Mais apoio pelas políticas públicas é necessário para ampliar o número de instituições credenciadas no país.

## INTRODUCTION

Brazil was one of the countries that participated in the policy meeting called *Breastfeeding in the 1990s: a Global Initiative*, promoted by the World Health Organization (WHO) and the United Nations International Emergency Fund for Children (UNICEF) in Florence, Italy at Spedale degli Innocenti in 1990. The main objective was to set global operational goals to promote, protect and support breastfeeding, as established in the Innocenti Declaration document, which complies with the International Code of Marketing of Breast-milk Substitutes and the implementation of the ten steps to successful breastfeeding.^1^

The Baby-Friendly Hospital Initiative (BFHI) was launched in 1991 in the United Nations’ member countries in order to ensure the practice of breastfeeding and the prevention of early hospital weaning. The document 10 Steps to Successful Breastfeeding^2^ establishes basic guidelines for a hospital policy that ensures the practice of breastfeeding. It consists of the mobilization and training of teams of professionals focused on the acquisition of the skills necessary for effective clinical and management practices for the promotion, protection, and support of breastfeeding.

Brazil was one of the countries selected to initiate the BFHI by signing the Innocenti Declaration, committing itself to making the ten steps to breastfeeding a reality in childbirth care centers. Currently there are more than 22,000 BFHI institutions distributed in 150 countries worldwide.^3^ BFHI is considered to be one of the components of a broader set of breastfeeding interventions, and its guidelines have been expanded to care for women and newborns in neonatal units. It also integrates the care network from the prenatal period to the outpatient follow-up of the child.

In Brazil, it is important that almost all deliveries take place in health facilities, which makes this strategy have a major impact on breastfeeding rates. Data from 2014 from the Information System of Live Births (*Sistema de Informações de Nascidos Vivos* - SINASC) registered 2,979,259 births performed in hospitals in Brazil.^4^ National and international studies show a positive association between births at Baby Friendly Hospitals and increased breastfeeding rates.^5^ ^,^ ^6^ ^,^ ^7^

The WHO guidelines are supported by a robust body of evidence indicating that breastfeeding behaviors are strongly linked to the reduction of morbidity and mortality for the child and mother both immediately at birth and throughout life.

For the mother, lactation is a factor that prevents breast and ovarian cancer and cardiovascular disease. The risk of postpartum hemorrhage is reduced when breastfeeding begins immediately after delivery.

For the child, breastfeeding provides the best nutrition, which is essential to reduce morbidity and mortality in the first years of life. It is also related to the intelligence quotient, as observed in a prospective cohort study in which a random sample of newborns at 30, 90 and 180 days of age was followed. In 560 eight-year-old children, an evaluation of their general intellectual capacity was performed using the Raven test. Breastfeeding for six months or more was associated with better performance in overall intellectual assessment, even after adjusting for the main confounding factors.^8^ Another important aspect of exclusive breastfeeding is the protective effects for children. Epidemiological studies have shown the beneficial effects of breastfeeding on the health of the child in the short and long term, with both mortality and morbidity decreasing.^9^ ^.^ ^10^

In 2017 BFHI completed 25 years in Brazil, a historical milestone in a program with a major impact on children’s health and part of the UN’s sustainable development agenda.^11^ However, over the years, the hospital titling process has not been uniform, with variations due to public policies related to the care of women and children. In spite of this, Brazil has proved itself in recent years to be an example with regard to the practice of breastfeeding, with its policy and strategy of assistance given to mothers and children, as well as through the BFHI. Progress has been made in guaranteeing the rights of children and women, in addition to qualifying comprehensive care in prenatal, childbirth, and postnatal care in the first two years of life.

This article describes the history of the BFHI from its implantation in 1992 until the year 2017, as a 25-year experience in Brazil.

## PUBLIC POLICIES AND BREASTFEEDING

Public policies in favor of women’s and children’s health have as their pillars the promotion, protection, and support for breastfeeding with an aim to improve the quality of life of children and women, the family, and the development of society. In Brazil, the first public health program focused on infant feeding was created in the 1940s, and was delegated to the National Department of Children of the Ministry of Education and Health, with the support of the Instituto Fernandes Figueira (IFF), however only starting in the 1980s were new policies implemented in Brazil.

Health care for children and women has been progressively regulated with direct and indirect benefits for breastfeeding. Considering this, the following can be cited: the National Breastfeeding Incentive Program (*Programa Nacional de Incentivo ao Aleitamento Materno* - PNIAM) in 1981; the Joint Mother and Child Accommodation in 1983; maternity leave of 120 days in 1988; the marketing standard for breast-milk substitutes and human milk banks in 1988; the Child and Adolescent Statute (*Estatuto da Criança e do Adolescente* - ECA) in 1990; the BFHI in 1992; the humanization of prenatal care and birth and care for low birth weight newborns - the Kangaroo Method -, from the Ministry of Health, in 2000. Federal Law No. 11,108 established the right for women to have someone accompany them during labor, delivery and immediate postpartum care in 2000, a milestone in the quality of perinatal care that ensures the family’s participation around birth.

The Ministry of Health’s Children’s Book, introduced in 2006, made it possible, among other things, to assess the risks for early weaning by recording information on maternal and neonatal factors around birth. One landmark was Law No. 11,265, published in 2006, which introduced changes in the commercialization of foods for infants and toddlers, nipples, pacifiers and bottles. It was called The Brazilian Standard for the Commercialization of Baby Foods and Early Childhood, Nozzles, Pacifiers and Bottles (NBCAL). It was an improvement over the previous regulation, published in Ministerial Order no. 2,051 / 2001, in the Resolution of the Board of Directors Collegiate (RDC) nº 221/02 and in the RDC nº 222/02, of the National Agency of Sanitary Surveillance (*Agência Nacional de Vigilância Sanitária* - ANVISA). In 2008, Law No. 11,770 was enacted, which extended maternity leave to 180 days within the public federal administration.

**Figure 1:**
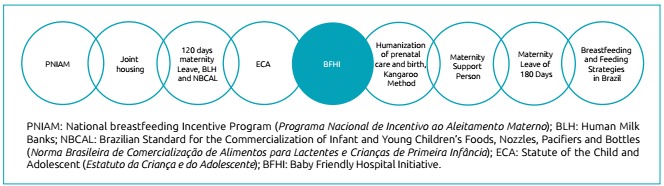
Timeline with programs, initiatives and Brazilian legislation to improve mother and child care.

More recently, the National Strategy for the Promotion of Breastfeeding and Healthy Complementary Feeding in the Public Health System (*Sistema Único de Saúde* - SUS) - Strategy for Breastfeeding and Feeding in Brazil -, regulated by Administrative Rule no. 1,920 / GM, published by the Ministry of Health in 2013, can be highlighted. It integrates the actions of the hospital component to outpatient care. The focus is to enable the promotion of breastfeeding practices in basic care. In this way, criteria were defined for the development of educational actions with regard to women’s rights and good practices during childbirth. The national achievements in maternal and child care described are presented in Figure 1.

## RULES AND PROCEDURES OF THE BABY FRIENDLY HOSPITAL INITIATIVE

The Innocenti Declaration, which served as the political and scientific basis for the origin of the BFHI, is a set of goals established for the recovery of a woman’s right to successfully breastfeed.^1^ It is based on the 10 Steps to Successful Breastfeeding, of the WHO and UNICEF, which are listed below:

- Step 1: Have a written breastfeeding policy that is routinely conveyed to the entire health care team.
- Step 2: Train all health care staff in the practices necessary to implement this policy.
- Step 3: Inform all pregnant women about the benefits and management of breastfeeding.
- Step 4: Help mothers initiate breastfeeding within the first half hour after birth.
- Step 5: Show mothers how to breastfeed and how to maintain lactation even if separated from their children.
- Step 6: Do not give newborns food or drink other than breastmilk, unless there is a medical necessity.
- Step 7: Practice together time - allow mothers and babies to stay together 24 hours a day.
- Step 8: Encourage breastfeeding at any time.
- Step 9: Do not offer artificial nipples or pacifiers to breastfed children.
- Step 10: Promote support groups for breastfeeding and refer mothers to these groups at the time of discharge from the maternity ward.

The implementation of the 10 steps in hospitals is guided by Brazil’s public health strategies and policies, which are based on WHO and UNICEF recommendations for good practices in childbirth care. The focus is on the success of breastfeeding and the overall health of women and children. It is the responsibility of the Ministry of Health to establish technical norms and guidelines for the habilitation of hospitals, in addition to determine the parameters that must be evaluated in the accreditation process. In the Ministry of Health, BFHI is under the administrative responsibility of the General Coordination of Child Health and Breastfeeding (*Coordenação Geral de Saúde da Criança e Aleitamento Materno* - CGSCAM), the Department of Strategic Programmatic Actions (D*epartamento de Ações Programáticas Estratégicas* - DAPES), and from the Secretariat of Health Care (*Secretaria de Atenção à Saúde* - SAS). Hospitals and maternities must complete 80% of all of the goals set by the BFHI both for adherence to and maintenance of the program.

In addition to complying with the 10 Steps to Successful Breastfeeding, it is necessary for public and private institutions interested in receiving the title and maintaining accreditation of the BFHI to comply with other requirements, redefined by Administrative Rule No. 1,153, of May 22, 2014, of the following items:

- Submit applications through the website www.saude.gov.br/crianca and fill out the forms that are available in the information system of the Ministry of Health.
- Comply with Law nº 11.265 made on January 3, 2006, and comply with NBCAL.
- Ensure free access to the mother and father and allow the mother or father to stay with the newborn for 24 hours, according to Ordinance No. 930, established on May 10, 2012. The hospital must have a written policy that is routinely transmitted to the entire care team.
- Include the overall criterion of Care for Women in the BFHI evaluation process. The hospital must have a written policy that is routinely transmitted to both the care team and the entire health care team.

The global criterion for Women Friendly Care requires the following practices:

- Ensure that pregnant women in their last trimester of pregnancy have a connection with the hospital where their delivery will take place.
- Assure that women may choose a companion to provide physical and/or emotional support during labor, delivery and the immediate puerperium.
- Offer women liquid and light foods during labor.
- Encourage women to walk and move about during labor, if they wish, and to adopt positions of their choice during childbirth, unless there are medical restrictions. This must be explained to them and conditions must be adapted to account for them.
- Guarantee women a quiet and cozy environment with privacy and soft lighting.
- Provide non-pharmacological methods of pain relief, such as a bath or shower, massagers/massages, a Pilates ball, hot and cold compressions - techniques that should be taught to the women during prenatal and parturient knowledge.
- Ensure care that reduces invasive procedures, such as ruptured membranes, episiotomies, acceleration or induction of labor, instrumental or caesarean deliveries, unless complications arise. If the procedures are necessary, they must be explained to the woman.
- If the hospital routinely has community / voluntary doula, authorize them and allow them to support the woman, if she desires.
- Ensure that mothers and newborns are responsible for hospital discharge and counter-referral in basic care, as well as access to other services and support groups for breastfeeding.
- Adopt basic care educational measures, from prenatal to puerperium, which aim to stimulate the Good Care Practices during Childbirth, from the WHO’s recommendations for care during normal childbirth.

Among other requirements, it is necessary for hospitals to have a cesarean surgery rate that is less than or equal to 30% or present a plan to reduce them by 10% per year, as specified by Ordinance No. 1,020, dated May 29, 2013, which establishes guidelines for the organization of health care in high-risk pregnancies.

**Figure 2:**
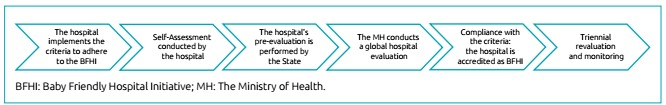
Steps for an institution to become a Baby Friendly Hospital.

Brazil is the only country in the world that requires compliance with these requirements and recognizes the importance of these aspects in the protection, promotion, and support of breast feeding.^12^ ^,^ ^13^ ^,^ ^14^ However, with the Ministry of Health’s redefinition of the inclusion criteria in 2001, 2004 and 2008,^15^ there was a slowdown in the accreditation of new health units in the BFHI. The procedures and stages for accreditation include: filling out forms and then a pre-assessment by the State Health Department, a global assessment by Ministry of Health accredited evaluators, and maintenance of the information system *web* (BFHI Information System - SIS-IHAC) for data collection and management. However, if the hospital cannot meet the requirements of steps 2 or 3, it can request a new evaluation in 90 and 180 days, respectively. After accreditation, the hospital should maintain a system to monitor the instituted policies in order to sustain the changes achieved. The State Department of Health promotes an annual reassessment, and the Ministry of Health encourages one every three years. Discrepancies may occur during monitoring of the process. In order to illustrate this, Figure 2 presents the steps for the institution to become BFHI.^16^

Regarding the training of professionals, the Ministry of Health recognizes the need for the continuing education of professionals involved in perinatal care. In 2008, courses were held in several states to train trainers and evaluators, raise awareness among managers, promote, support and manage breastfeeding, and provide information about NBCAL. In 2009, with the action of the Pact for the Reduction of Infant Mortality in the Northeast and in the Legal Amazon, workshops were organized for 425 managers at 147 maternity hospitals in those regions on the subject of BFHI. In 2010, the actions took place in the Southern, Southeastern and Midwestern states for 152 managers from 45 hospitals and maternity hospitals.^15^ In order to complete the BFHI, all of the professionals of the hospital unit should attend training courses.^16^

## A BRIEF HISTORY OF THE EVOLUTION OF THE BABY FRIENDLY HOSPITAL INITIATIVE IN BRAZIL

The Instituto Materno Infantil de Pernambuco (IMIP) was the first hospital in Brazil to receive the title of Baby Friendly Hospital in 1992. This is a remarkable fact, because it was the first to meet all of the criteria for accreditation and to demonstrate that it is feasible for a Brazilian institution to become a Baby Friendly Hospital.

Accreditation of hospitals has occurred irregularly over the last 25 years. The second accredited institution was the Hospital Guilherme Álvaro, which is linked to the Department of Pediatrics at the School of Medical Sciences in Santos. It should be noted that these two hospitals played an important role in the training of multi-professional teams that started the accreditation movement of new hospitals. Several actions, such as courses and the training of teams to encourage successful breastfeeding, have contributed to modify ways of working in different regions of Brazil.

Between 1992 and 2010, 359 institutions were accredited and 26 hospitals were disqualified in Brazil. After 2005, the number of accessions slowed down due to the difficulty in meeting the requirements. In 2010, 18 more hospitals were disqualified. In 2008 there were 337 accredited institutions, distributed as follows: 153 in the Northeast, 72 in the Southeast, 54 in the South, 37 in the Midwest and 21 in the North.^17^

In 2014, the number of Baby Friendly Hospitals in Brazil was 323, with the most in the Northeast Region and the least in the North Region. In 2015, the Ministry of Health registered 326 accredited hospitals. Even with variations in the number of institutions, Brazil is still considered one of the countries with the highest prevalence of exclusive breastfeeding in the world. In the period between 2006 to 2013, breastfeeding rates increased. From 1986 to 2006, the prevalence of exclusive breastfeeding at 6 months in children under 2 years and continuing up to 1 year of age increased from 4.7, 37.4 and 25.5% to 37.1, 56.3 and 47.2%, respectively.^18^ This result can be partly attributed to the work processes developed in the BFHI.

## DIFFICULTIES FOR THE IMPLEMENTATION OF THE BABY FRIENDLY HOSPITAL INITIATIVE

The lack of motivation for changes in care practices is an important factor identified in some accredited hospitals.^19^ In order to comply with the ten steps, it is necessary to integrate the points of attention in the care network, with support from prenatal care to hospital care. In hospitals, the commitment of health professionals and the autonomous definition of the mother must be included. The ten steps need to be followed for the success of breastfeeding and this depends on both the mothers and the health team. A study on the evaluation of accredited hospitals showed that the difficulty in implanting it was related mostly (80%) with steps 6, 7 and 9. Next were steps 3 and 5, with about 70%; in steps 1, 4, 8 and 10, less than 50%.^20^ In another investigation, non-compliance with step 6 was linked to the use of dairy supplements. In this case, the risk of reducing exclusive breastfeeding between 30 and 60 days and stopping breastfeeding at 60 days was two and three times higher, respectively.^21^

In a study carried out in Recife at five accredited hospitals, 419 mothers were interviewed to evaluate the implementation of the 10 Steps to Successful Breastfeeding. The inclusion criteria were mothers who underwent prenatal and delivery at the research institutions and postpartum for more than 6 hours, did not have clinical complications during their delivery and immediate postpartum, and gave birth to low risk newborns. The results showed that steps 3 and 5 were below recommended levels (<70%), and steps 1, 4, 8 and 10 were also lower than the limit established by the BFHI (<50%). Only steps 6, 7 and 9 were performed in more than 80%. The results reaffirm the need to intensify actions to monitor adherence to the ten steps, in addition to monitoring the criteria established in the BFHI.^22^

In the operation of the BFHI, qualitative evaluations can contribute with important information regarding how the care teams’ work processes are organized.^23^ In a review study, the impact of breastfeeding training on the knowledge and practical skills of the professionals was identified. Among the 17 articles that matched the methodology in the systematic review, all forms of training had a positive impact on knowledge, skills and/or professional and hospital practices.^24^

## THE IMPLICATIONS OF THE BABY FRIENDLY HOSPITAL INITIATIVE ON THE SUCCESS OF BREASTFEEDING

BFHI has been associated with improved rates of exclusive breastfeeding and regular breastfeeding.^25^ ^,^ ^26^ The importance of breastfeeding for child growth and development in addition to women’s health has been proven through scientific studies.^10^ Breastfeeding is able to reduce the number of deaths due to preventable causes of children under 5 years of age by 13% in the world. Furthermore, breastfeeding helps reduce chronic diseases such as hypertension, obesity and diabetes mellitus when the children become adults.^9^ ^,^ ^28^ It also decreases the risk of breast and ovarian cancer and type 2 diabetes mellitus in breastfeeding women.^27^ The positive effect of BFHI on increasing breastfeeding rates has the direct consequence of short- and long-term benefits for the mother and child.^9^ ^,^ ^10^

The 10 steps for the implantation of the BFHI at the Regional University Hospital of North Paraná were measured before and after the accreditation. There was a significant increase in the exclusive breastfeeding index, which reached around 95%. The success has been attributed to a sum total of efforts from the whole team, with changes in behaviors and attitudes regarding the promotion, protection, and support of breastfeeding. Integrating the different sectors involved in care was important to create harmony among the teams. The results showed that the strategy triggered considerable changes in the practice of breastfeeding while the mother was hospitalized for childbirth. The initiative contributed to stopping the use of milk formulas.^29^

National research found that healthy infants born in Baby Friendly Hospitals were less likely to have unnecessary interventions soon after delivery, such as aspiration of the airways, use of inhaled oxygen, and use of an incubator. Skin-to-skin contact with the mother soon after birth, breastfeeding in the first hour of life while still in the delivery room, and joint housing were more frequent in these institutions. The authors conclude that Baby Friendly Hospitals are a reference in the quality and humanization of care during all stages of gestation, delivery, birth and the early neonatal period.^30^

Successful breastfeeding is related to several factors. Among them, the short period of hospitalization hinders the mothers’ ability to perform exclusive breastfeeding, especially primiparous mothers. At home, pressure coming from grandparents to start weaning and introducing other foods to the child, are part of reality. Therefore, it is fundamental to work with the clinical processes that are integrated in the network, starting from the prenatal period in the maternity reference health units. Actions in permanent education with mothers and health professionals should be supported by managers, in a policy that is favorable to breastfeeding. A good opportunity to do this is through the dissemination of this knowledge to the basic care units which are associated with maternity hospitals.

## CONCLUSIONS

The experience accumulated during these 25 years of BFHI in Brazil has shown that the number of institutions accredited by SUS is still small - in 2013 it was 5,530 hospitals - considering the territorial dimension of the country and the large number of existing maternity hospitals.^31^

Advances have been slower than expected and may be related in part to difficulties in meeting the criteria currently set by the Ministry of Health. This study may contribute information to make necessary improvements and adjustments in policy that encourages and supports BFHI in Brazil.
